# Military Dogs and Their Soldier Companions: The More‐than‐human Biopolitics of Leishmaniasis in Conflict‐torn Colombia

**DOI:** 10.1111/maq.12694

**Published:** 2022-02-02

**Authors:** Lina Pinto‐García

**Affiliations:** ^1^ Department of Science & Technology Studies York University Toronto Ontario Canada

**Keywords:** *nonhuman*, *improvised explosive devices*, *landmines*, *affect*, *zoonosis*

## Abstract

Cutaneous leishmaniasis is a vector‐borne disease that produces growing skin ulcers. In Colombia, the transmitting phlebotomine sandfly is native to the same jungles that have been the primary theater of war. Although combatants are the most affected by leishmaniasis, military landmine detection dogs are also significantly impacted. This article draws on ethnographic field research with human and canine members of the Colombian military. While their leishmaniasis ulcers constitute a shared expression of violence that makes evident the closeness of the human–dog bond, differences in their state‐provided health care reveal the production of shifting species hierarchies. I argue that war scrambles both human**–**dog affective relationships and biopolitically configured interspecies hierarchies in ways that produce suffering, not just for humans and dogs separately, but also for the bonds they forge together. Building peace through health care demands repairing the ways in which armed violence has rendered the bonds between humans and nonhumans pathological.

## Introduction

By the end of 2016, Julián^1^ had recently turned 24 and completed six years as a professional soldier in the Colombian army.^2^ He had spent most of that time in the southern part of the country, participating in military operations within densely forested tropical environments. This work made Julián vulnerable to phlebotomine sandfly bites and the disease these tiny insects transmit in these peripheral landscapes—cutaneous leishmaniasis.^3^ This is a noncontagious, nonfatal parasitic skin disease that starts out like a tiny sore and continues to grow into an ulcer. Although usually painless, a leishmaniasis lesion often requires pharmaceutical treatment to scar over. Julián's ulcer was located on his left leg, right where the boot rubs against the skin, which caused it to grow rapidly. When we met, he had just started 20 days of antileishmanial therapy, and I had just began my ethnographic research on the relationship between leishmaniasis and the Colombian armed conflict.

Julián was part of a human–dog pair known in the army as a *binomio canino* (canine binomial). The second half of that virtually inseparable duo was Lluvia, a black female Labrador dog that the army assigned to Julián. The binomial's job is to detect landmines and other improvised explosive devices hidden in the rainforest and other rural areas of Colombia. The hope is that this will prevent the troops from triggering an explosion that can disrupt military operations by producing injured, maimed, disabled, and dead bodies. Before entering into the Leishmaniasis Recovery Center (CRL)—a military clinic dedicated exclusively to treating army personnel with leishmaniasis—Julián had to leave Lluvia with his battalion veterinarian. The disease had also appeared on the dog's nose while they were together in the area of operations. “That little dog must be going crazy without me. She can't live without me, and I can't live without her,” he said to me.

At the time of that conversation, I knew that leishmaniasis also affected dogs. However, based on my readings of scientific papers, I used to think of canine leishmaniasis mostly as a public health problem in southern European countries (e.g., Miró and López‐Vélez 2018). I had not imagined this was an issue in Colombia, let alone a significant problem for anti‐explosive dogs of the Colombian army. Although I was aware of and attuned to the multiplicity of species participating in the phenomenon biomedicine calls “leishmaniasis,” when I realized that military dogs were also bitten by infected sandflies and developed ulcers in the context of war, leishmaniasis appeared to me as an *animal inclusive disease* that is contingent on a cast of living beings that could not be predetermined or taken for granted (Nading 2013).^4^ I therefore felt compelled to make sense of this perplexing realization—what Agar (1994) would call a “rich point”—by seeking access to one of the 18 military Canine Training and Retraining Centers (CERCAs) where I could explore some of the sociocultural aspects of canine leishmaniasis in the context of the Colombian conflict.

Through ethnographic field research conducted between 2016 and 2017 within the CERCA of the Liborio Mejía Battalion (Florencia, Caquetá) and the CRL (Duitama, Boyacá), leishmaniasis gave me a powerful lens to understand war as a more‐than‐human phenomenon that scrambles both interspecies affective bonds and biopolitically configured interspecies hierarchies. As such, this article shows that war reworks the affective and biopolitical dimensions of the human–dog relationship, producing interspecies reverberations of state violence that usually remain in the shadows and well beyond the purview of peace‐building efforts.

In developing this argument, I first show that war confounds interspecies hierarchies in ways that, whether anthropocentric or zoocentric, produce forms of suffering not only for humans and dogs separately, but for the bonds they forge together. Second, I contend that, despite the fundamental role that affect and empathy play in the dog–soldier relationship, as well as in the collaborative task the military expects them to carry out, these interspecies attachments tend to be dismissed and sacrificed in the name of war. In this case, the problem is not a lack of interspecies compassion guided by anthropocentrism, but the broader framework of power and violence where soldiers, dogs, and their loving relationships are kept alive and strong only as long as they are instrumental to the war and its continuation. Last, I highlight the need to consider interspecies health as a peace‐building domain in Colombia's highly fragile post‐conflict. In particular, this involves attending to animal suffering in the context of war and repairing the ways in which armed violence and militarization have rendered the bonds between nonhumans and humans pathological.

## Biopolitics and the More‐than‐human Ontology of Health, Disease, and War

Traditionally, medical anthropology has dealt exclusively with human health. Yet, ethnographic explorations of animal health, especially in light of the ongoing COVID‐19 pandemic, have gained significant importance, given the pressing need to understand how human proximity and contact with wild and domestic animals shape health, disease, and healing. Brown and Nading (2019) have made the case for a more‐than‐human understanding of health to destabilize disciplinary settlements, but also to foreground the expansion of biopolitics beyond the human and interrogate the ways in which care practices reinforce, dissolve, or redraw boundaries between species. While public health often seeks to reduce or eliminate human–animal contact to avoid pathogenic interactions, these efforts usually fail because they overlook “the depths, intensities, and affective complexities of social relations between humans and animals” (Brown and Nading 2019: 6). Thus, shifting attention to relational aspects of interspecies contact exposes the incompleteness of conventional public and global health approaches to make sense of and address health problems involving nonhuman animals.

In a similar vein, Blue and Rock have introduced the concept of *trans‐biopolitics* to analyze how “contemporary biopolitical formations implicate human and nonhuman bodies in webs of complex relations with implications for a broader politics of health” (2011: 358). By focusing on the contextual ways in which power operates across boundaries between species, trans‐biopolitics is attentive to cross‐species interactions involved in human and animal health, how they change over time and space, and the distinctions made between different species through processes of governance.

This article draws attention to these biopolitical mechanisms as they take shape in the canine binomial, a technical idiom coined by the Colombian armed forces to name the pair constituted by a human and a trained dog employed in military, police, and humanitarian operations. Beyond its mathematical meaning, in Spanish, the word “*binomio*” indicates a set of two persons or things taken as a unit or as elements in equilibrium or dependent on each other.^5^ As Pardo Pedraza (Forthcoming) suggests, the “glue” that unifies and harmonizes the canine binomial is affect. Thus, military dogs and humans constitute cyborgs—chimeras established through an affective and repetitive training process where the dog, the human, and their co‐laboring relationship are made and remade through constant and mutual attunement. For Pardo Pedraza, the well‐known phrase from Haraway's *Cyborg Manifesto* (1985: 96)—“one is too few, but two are too many”— aptly describes this bi‐species formation born of war.

In this article, I adopt an affect‐inflected trans‐biopolitical lens to analyze those relational aspects between soldiers and military dogs resulting in and emerging from leishmaniasis, which highlight their biological commonalities and shared vulnerability in a conflict setting. As Livingston and Puar (2011) have pointed out, hegemonic ideas of human singularity and exceptionalism often establish intellectual, regulatory, and material hierarchies where humans are ranked above animals. In response, there is a growing scholarly interest in animality that, originally inspired by Haraway's (2008) influential work, challenges anthropocentrism to level the playing field so that humans are no longer assumed to be superior to nonhuman beings and cease to be the dominant object of analysis in political and social worlds. But is it true that whenever the human is decentered in practices of care, equity is gained? Is it possible that posthuman proclivities for care may lead to unwelcome consequences that deepen suffering when, for example, they are appropriated by a state at war? Drawing inspiration from the work of Ticktin, I am interested here in “forms of noninnocent care, which acknowledge the ways that care partakes in valuations of life” (2019: 137) and generates shifting, at times even animal‐centered, but still perilous distinctions and stratifications between humans and nonhumans.

An example of this, akin to what this article explores, is Hediger's (2013) biopolitical analysis of the use and subsequent abandonment of dogs by the U.S. military in the Vietnam War. In his view, a key paradoxical aspect of Foucaultian biopolitics is that “life is both more rigorously organised and controlled under biopower, and more entirely threatened” (Hediger 2013: 57). In a war scenario, he argues, this characteristic appears particularly intense and evident in the regularity and fluidity with which categories such as “human,” “animal,” and “machine” change value and shift hierarchical position. As a result, military dogs employed in Vietnam went from being celebrated as war heroes to being abandoned to their fate as disposable machines, sparking outrage in their military human companions.

Besides the trans‐biopolitical lens, this article adopts a conceptualization of war that attends to its *becomings*, while moving away from static or ready‐made definitions (Bousquet et al. 2020). By embracing an “ontology that is consonant with the confounding mutability of war” (Bousquet et al. 2020: 100), I am interested in war's more‐than‐human nature and, in particular, in the ways in which it produces and is waged through spaces and relations where soldiers and military dogs—but also sandflies and *Leishmania* parasites—are forced to become *enmarañados*, or closely entangled (Pinto‐García 2020). In other words, this article instantiates that war is a more‐than‐human phenomenon in which human–nonhuman relations can acquire pathological overtones. Under this perspective, leishmaniasis “is not so much ‘at the door’ but *incubated* through” the ways in which humans, animals, microorganisms, and environments relate to one another in the daily making of war (Hinchliffe et al. 2013: 539, emphasis in the original).

This understanding contrasts with the ways in which armed conflicts have conventionally been viewed—a human domain where the roles of perpetrators, victims, and witnesses belong exclusively to humans. Increasingly, however, anthropology and STS scholars are drawing attention to the intricate socioecological relationships between humans and nonhuman entities in contexts of militarized violence, which play a crucial role in warfare, as well as in the suffering and lasting consequences it entails (Kosek 2010; Lederach 2017; Pugliese 2020; Ruiz Serna 2017). By adopting a relational approach, these works provide an understanding of war that builds on—but also goes beyond—the ordinary limits of anthropocentric depictions of armed conflicts and their peaceful resolution (Lyons et al. 2019). For example, Dewachi (2019) has studied an antibiotic‐resistant bacterium that U.S. military surgeons called “Iraqibacter” to explore the biological and morbid legacies of war in the Middle East. He uses the notion of *war ecologies* to highlight the ways in which war generates the sociopolitical, historical, material, and environmental conditions necessary for the establishment of pathological relationships between microorganisms and humans.

Building on these understandings of the more‐than‐human nature of war, health, and disease, in what follows I use leishmaniasis as an entry point to illuminate the militarized and affective bonds that war creates and nurtures—but also compromises—between human and canine members of the army. I also trace the biopolitical dispositions through which a state at permanent war manages human and canine populations with leishmaniasis, disrupting affective relations and generating shifting stratifications within and across human and nonhuman categories. In particular, I highlight the various ways in which the wartime medical management of humans and dogs perpetuates anthropocentric forms of speciesist stratification, but also unsettles and even turns them upside down. Also, I interrogate the biopolitics that organizes hierarchically the control of leishmaniasis in populations of soldiers, civilians, and dogs, as well as the ways in which these nonhumans and the interspecies relations generated in a context of war challenge such control.

## Canine Leishmaniasis

For more than 50 years, Colombia has endured a bloody armed conflict involving state soldiers, far‐left guerrillas, and far‐right paramilitaries. Although a peace agreement was signed in 2016 between the government and the oldest and largest guerrilla group—the Revolutionary Armed Forces of Colombia (FARC)—violence continues, and it is inaccurate to speak of the current times in terms of *post*‐conflict (Gutiérrez Sanín 2020). After Afghanistan, Colombia holds the second highest number of landmine victims. Despite their prohibition in 1999 by the Ottawa Convention, the use of these improvised explosive devices, mainly by guerrilla groups, has been a prominent feature of the Colombian armed conflict to this day and a major challenge to peace‐building endeavors (CNMH and Fundación Prolongar 2017).

In the 2000s, Álvaro Uribe's government launched an unprecedented military offensive against guerrillas and increased recruitment by 31.6% (Leal Buitrago 2011). Numerous soldiers entered the rainforest and, for the first time, stayed there for several months at a time to maintain sustained military pressure against guerrillas, especially the FARC. Guerrillas responded with the massive use of landmines. In that period, battlefield injuries were not the major causes of army casualties. In fact, the harm caused by landmines and leishmaniasis was much more significant, leading to the annual withdrawal of approximately 10,000 people from military duties both in 2005 and 2006 (Pinto‐García 2020). Since those acute years of the war, dogs became crucial to tackle the threat posed by landmines to soldiers and military operations. So‐called explosives and demolitions groups (EXDE) were established to protect the military troops by finding and destroying explosive artifacts during field operations. An EXDE group includes a commanding sub‐officer, three soldiers, and a canine binomial (CNMH and Fundación Prolongar 2017). Although other animals such as pouched rats (DeAngelo 2018) and bees (Kosek 2010) have also been recruited and bred for military and humanitarian purposes elsewhere, dogs remain the main animal sensors of chemical explosives both globally and in Colombia.

Mine detection works through embodied communication between a soldier and a dog, and the joyful association the dog is trained to make between a ball and the smell of explosives when the soldier invites the dog to play. Julián explained to me that when he or any other member of the troop notices “anomalies” in the jungle—a pruned tree trunk, a tiny piece of plastic or paper, traces denoting someone has slept in that location, footprints, etc.—Julián stops walking and stands facing the area he wants Lluvia to examine. He hides the ball, taps his chest a few times, points his arm toward the zone of interest, and gives a command to Lluvia indicating that it is time to play. She runs in the direction indicated by Julián. If she detects the scent of explosives, she looks at him, then looks at the place she wants to signal, and sits. While Julián was telling me this, he imitated the dog's movements with his body and allowed me to visualize this more‐than‐human war routine practiced by several binomials, at any given time, in multiple parts of rural Colombia.

Jaime Rivera is one of the officers with a leading role within the National Center against Explosive Devices and Landmines (CENAM), the military department in charge of army dogs. For him, the FARC's use of mines became systematic after 2002. This turned dogs both into key members of the army and crucial actors of the war. According to Jaime, at that time, mines accounted for 40–60% of the casualties within the military, and the institution came to own 4,000 canine members. “The dog became very important, almost more important than the soldier himself,” he said.

The work that the war imposes on soldiers and dogs forces them to penetrate the jungle and to be easily bitten by sandflies. As a result, military dogs have been made into just another source of blood for leishmaniasis‐transmitting sandflies. In Jaime's view, 2008 is the year when leishmaniasis became a major health problem for army dogs. “Of the 3,000 dogs the army currently [2016] has, between 100 and 200 are affected annually by leishmaniasis,” he said. Their lesions are typically located in body areas with less hair—usually on the nose and the genitalia, and less often on the ears and the feet (Vélez et al. 2012)—and end up compromising dogs’ scent‐tracking skills and expected performance in military operations.

Grove's work (2016) is useful to make sense of the relationship between improvised explosive devices and canine leishmaniasis. He came to realize that the power of these devices lies in their volatile materiality, which makes it impossible to (pre)determine not only what these technical objects are but what their effects will be. Actually, their effectiveness can only be analyzed in relation to the contingent relationships that are created, intensified, and transformed in a specific environment and time. As such, it is more appropriate to speak of *emergence* rather than landmines’ causality or effect. Among countless other beings, Colombian jungles are populated not only by sandflies and *Leishmania* parasites, but also by combatants and landmines. The latter have also made dogs regular inhabitants of these conflict zones. Thus, canine leishmaniasis is a biosocial phenomenon that, in an unanticipated way, *emerges* from the widespread employment of landmines that characterizes the last decades of conflict in Colombia. Dogs bearing leishmaniasis ulcers and scars are yet another dimension of the unexpected effectiveness of landmines and their multiplicity in their intra‐actions with both humans and nonhumans.

In this context, the permanent condition of war and the extended employment of landmines have generated *borderlands* (Hinchliffe et al. 2013)—i.e., spaces where soldiers, dogs, and parasite‐carrying sandflies converge in the rainforest and produce leishmaniasis—in a simultaneous and relational way—among nonhuman and human members of the military. Moreover, the progression of warfare and dealing with the proliferation of landmines depend crucially and perversely on those affective interspecies interactions that simultaneously epitomize the conditions of pathogenic possibility in dogs and soldiers. In other words, the violence of war exploits the densities and intensities of cross‐species affective relationships. War perverts these bonds in such a way that disease is incubated through the biological commonalities, shared vulnerability, and loving collaboration that define the work and nature of the binomial.

## Leishmaniasis Treatment across Species

In Colombia, leishmaniasis typically occurs in densely forested tropical environments where sandflies thrive. These jungles constitute the main theater of war. Not surprisingly, state soldiers, paramilitaries, and guerrillas form the demographics most affected by leishmaniasis (Patino et al. 2017). As I explore elsewhere, this close relationship between leishmaniasis and the conflict has led to the stigmatization of leishmaniasis as “the guerrilla disease” and altered the circulation of and access to the standard treatment—Glucantime—as a war strategy to affect guerrillas (Pinto‐García 2020). Soldiers have had privileged access to this drug and to specialized health care. By contrast, the rest of the population has faced significant access barriers to antileishmanial drugs, shaped both by the conflict and the market‐based structure of the health care system. While marginalized people in remote and conflict‐ridden areas of Colombia endure violence when denied access to Glucantime, soldiers also suffer significant harm when the only treatment that public health chooses to offer is a highly toxic, old, and often ineffective pharmaceutical (Pinto‐García 2021). This is especially poignant considering that these are mostly uneducated young men, from poor and often rural families, who often enlist in the army out of necessity or obligation.

Regarding the pharmaceutical care of military dogs with leishmaniasis, there was a significant change around 2014. Before that, they used to be treated with the same drug as soldiers (Glucantime).^6^ From the large stock of this drug that the Ministry of Health (MinSalud) purchases and sends to the army's Health Office, the latter used to allocate a small stock for any of the CERCAs that reported cases of canine leishmaniasis.

Thus, like soldiers, army dogs used to have better opportunities for access to Glucantime than civilians and guerrillas affected by the disease in rural areas of the country. Put another way, the pharmaceutical needs of nonhuman military members used to be better served than those of nonmilitary human populations in rural areas of Colombia. Insofar as they are made into mine detectors and protectors of military personnel's lives, army dogs used to take priority over civilians affected by the disease. This stratification reveals a war logic that places more value on nonhuman lives that are key to the perpetuation of the conflict than on the human lives of the most marginalized in society.

However, things changed around 2014. Although the Glucantime delivery system to meet the demand of military dogs suffering from leishmaniasis had been in place for years, the army interrupted it abruptly. At that time, someone alerted the army that the drug purchased by MinSalud was only authorized for use on humans, not on dogs or other animals. To avoid the development of drug cross‐resistance in *Leishmania* parasites infecting dogs and humans, MinSalud followed the World Health Organization's recommendation to reserve antileishmanial pharmaceuticals used in humans for treating humans only and not for veterinary purposes (WHO 2010: 949). As all Glucantime ampoules legally available in Colombia must be purchased, imported, and distributed exclusively by MinSalud, this meant that all dogs affected by leishmaniasis—army dogs included—were suddenly left in a therapeutic limbo with no pharmaceutical treatment.^7^ Thus, since 2014, army dogs have not been treated with Glucantime or any other antileishmanial pharmaceutical product. Trying to provide a therapeutic alternative, military veterinarians have reviewed the scientific literature and found that other pharmaceuticals—allopurinol, mabofloxacin, and ketokonazole—might be useful to eliminate (leishmanicidal effect) or at least inhibit the growth (leishmanistatic effect) of *Leishmania* parasites in dogs. Unlike Glucantime, these drugs do not have any sort of restriction and are not used to treat leishmaniasis in humans. Thus, the army can easily purchase them independently from MinSalud. However, the results have not been encouraging. During my fieldwork, there were numerous dogs whose lesions resisted healing despite these treatments.

The changing access that army dogs have had to antileishmanial drugs points to shifting biopolitical distinctions between humans and dogs in Colombia's (post‐)conflict. At times, military nonhumans (like military humans) ranked above civilians and guerrillas, revealing a preference for those in a security and defense role by a state at war. In more recent times, however, military dogs have lost this superiority and are ranked below soldiers, as civilians, guerrillas, and nonmilitary dogs affected by leishmaniasis have always been. This loop of distinctions within and across human and nonhuman categories indicates that decentering the human does not necessarily guarantee a more even playing field. In fact, it shows that war is a perverse phenomenon, which establishes differential ways to exert violence on multiple life forms and breeds injustice in one way or another depending on its needs.

Significantly, this provides an important counterpoint to the work of anthropologist Uribe Alarcón (2018), who has studied massacres and how this form of violence shows continuities from the mid‐20th century to the present in Colombia. Uribe Alarcón argues that animalization has functioned as a metaphor for domination in Colombia. For her, animalizing the other—blurring the human/animal divide—allows perpetrators not only to suspend the taboo against killing fellow humans within a very Catholic society, but also to degrade and dehumanize the victims by turning their death into a mere practice of guilt‐free butchery. But here we are dealing with a different combination of violence and animality, where the animalization of the human does not facilitate violence against humans. Rather, what is at issue is on whom it is permissible to exercise violence. The subject of greater protection is not always the human, but the one whose life takes precedence according to the needs of war—it is a violent shifting hierarchy across and within species.

## Challenging Public Health Policies through Affect

In addition to the logistical problem that all canine leishmaniasis cases present to army operations, these dogs, as well as those who are asymptomatic,^8^ also constitute a challenge in terms of public health. Since they turn out to be potential reservoirs of the disease—animals in which *Leishmania* parasites live and multiply and can be transmitted to humans and other dogs in the presence of sandflies—all dogs with leishmaniasis are also considered a public health concern (Beiter et al. 2019).

In December 2018, I submitted an access to information request to MinSalud inquiring about the management of army dogs with leishmaniasis. Omitting the earlier use of Glucantime in nonhuman army populations, my question was replied to in the following way:

Medicines purchased by the Ministry of Health for the care of patients suffering from leishmaniasis are registered for human use, therefore, the medicines that have been assigned to the army are for the treatment of humans, not for canines.

I also asked about MinSalud's strategy to address the public health problem posed by dogs infected with *Leishmania*. Reiterating the prominence of euthanasia in the institutional response to zoonoses (see Hurn and Badman‐King 2019), this was the reply:

According to what is established in international standards, which apply to our country, canine reservoirs with a positive diagnosis for leishmaniasis, because they constitute a risk in terms of public health, are subjected to canine control through euthanasia under the consent of the owners.

**Figure 1:**
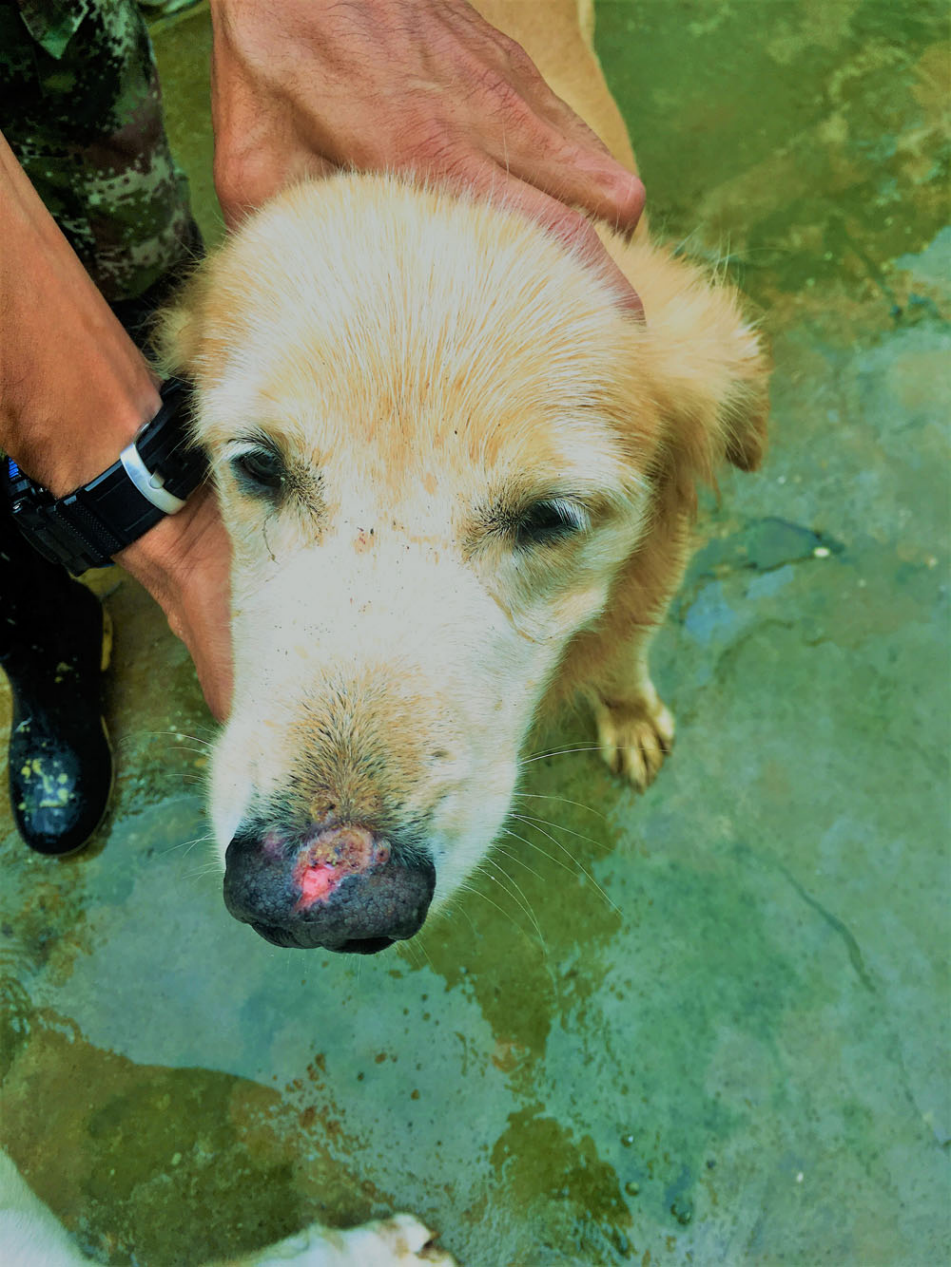
Soldier showing the leishmaniasis lesion of his dog coworker. Photo by the author. [This figure appears in color in the online issue]

For army dog handlers, however, euthanizing military dogs with leishmaniasis feels like a moral and affective injustice. The emotional bond that unites soldiers like Julián and dogs like Lluvia is one of deep affection, incompatible with a public health provision that seems to handlers grossly disproportionate. For many soldiers, dogs are not simply companions or co‐combatants; in their words, they are their daughters and sons (see Figure 1). Military veterinarian Gustavo Fuentes thinks that culling military dogs because of leishmaniasis is “an extremely unfair measure,” especially after the animal “has provided such a valuable service” preserving the life of thousands of soldiers. “In that case, humans [with leishmaniasis] should also be killed because they too are reservoirs of the disease,” he said, stressing how absurd he thought it was to kill a dog because it potentially represents a source of parasites. Doing everything possible to treat a dog and get it to overcome leishmaniasis is, for Gustavo, just a minimal moral obligation. “We must give dogs a chance and, in that way, thank them to some extent,” he said. Also, he thinks that killing a dog who has been selected, trained, and retrained to work as a living explosive detector represents a major waste of money and time for the army.

The affective and emotional ties between soldiers and dogs challenge the instrumental rhetoric that rationalizes culling in the governance of diseases capable of crossing barriers between animals and humans (Blue and Rock 2011). By indicating that humans can also be understood as reservoirs of the disease, Gustavo was drawing attention to the hierarchies between dogs and humans that often operate in the public health management of zoonoses. He was also questioning the politics of death imbued in public health policies that justify killing dogs to preserve human life, especially in a war context where dogs become sick while serving the needs of a state—and a society—that employs them to protect human life and military performance. As such, dogs’ key involvement in warfare, by way of building close bonds with humans, provides dogs with a position from which to shape the political and challenge public health dispositions. While it is undeniable that human discourse and agency in the context of warfare far outweigh the influence a dog can have on decision‐making at the level of public policy and state institutions (Srinivasan 2013), this also instantiates how “processes of interspecies exchange and affective transit **…** work through yet leak beyond structural organization of human agency and cognition” (Ahuja 2016: xiii).

In conversations with military dog handlers and trainers, I asked them what they thought was worse, a dog or a soldier with leishmaniasis. “It's the same!” is the reply I consistently obtained. As dogs and soldiers with leishmaniasis have to be evacuated from the area of operations, their absence undermines military operations in crucial ways. Esteban Cruz, a professional soldier who had been part of the army for 17 years, 14 of them working with dogs, described this situation as follows:

If you don't have the soldier, the soldier is missed, and if you don't have the dog, the dog is missed. Why? Because both are indispensable. The dog can't be alone, and the soldier can't do the work the dog does. Neither of the two can perform on its own. The soldier doesn't have the olfactory ability of the dog, and the dog can't work without the person who guides him/her. They need to be two.

In Esteban's view, leishmaniasis is one of the customary ways in which dogs and soldiers—as well as the binomial they form—suffer from war. For him, dogs are victims because they go through the same hardships and sufferings soldiers go through in the area of operations. “They fall into explosive devices, lose their lives, are attacked by enemy fire, and get leishmaniasis; if there were no war, we wouldn't have to use dogs,” Esteban said. It is the conjoined bodily experience of gunfire, mines, and leishmaniasis that puts dogs and soldiers on a similar plane, revealing their shared vulnerability, biological commonalities, and coupled victimhood. As much as they are war actors and far‐from‐innocent counterinsurgent combatants, dogs are also victims because, like poor and marginalized young men in Colombia, they are “forced into ‘becoming with’ [a warfare] state apparatus” (Haraway 2008: 37).

## Serena

**Figure 2:**
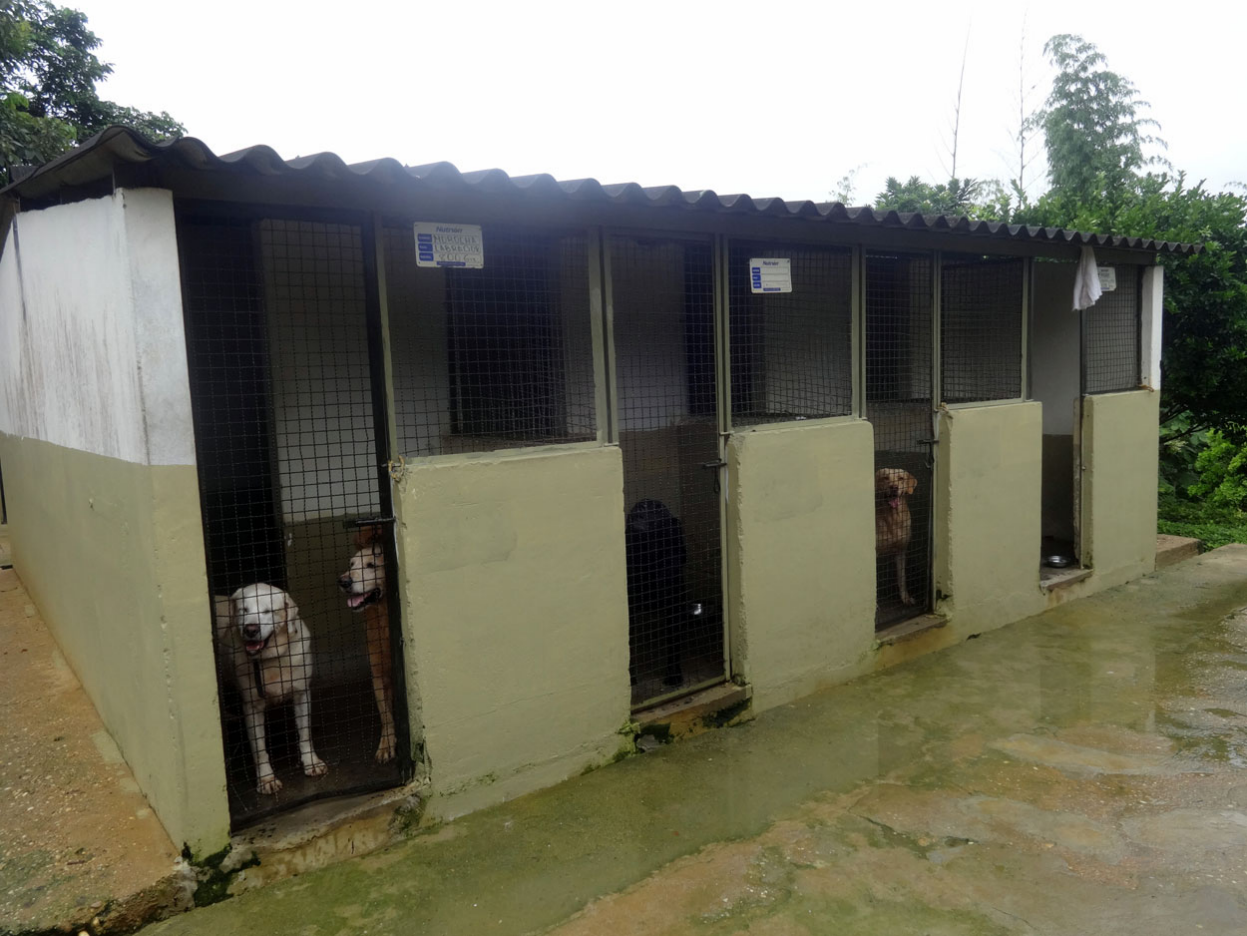
Some of the kennels at the Liborio Mejía Battalion's CERCA. Photo by the author. [This figure appears in color in the online issue]

**Figure 3:**
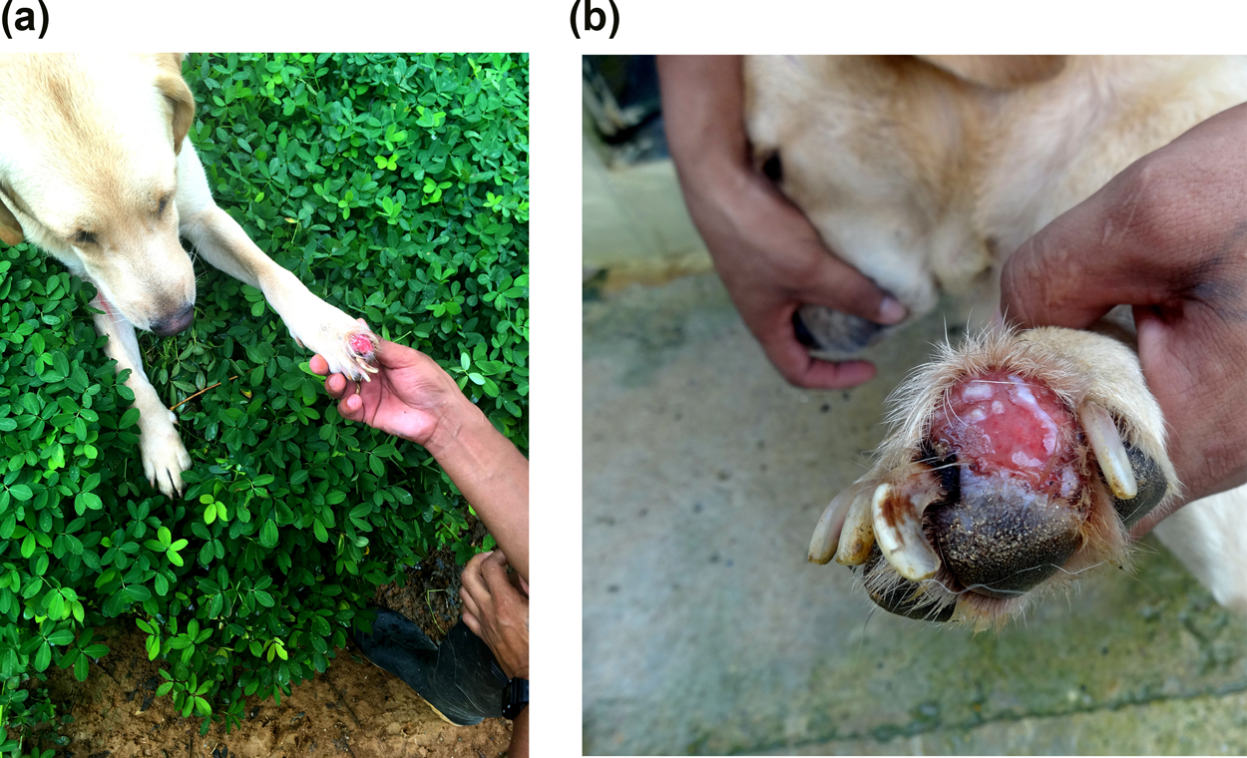
(a) Serena, her leishmaniasis lesion, and her soldier companion. (b) Close‐up of Serena's leishmaniasis ulcer. Photos by the author. [This figure appears in color in the online issue]

While soldiers were cleaning up the military canine training center, most dogs were kept tied up or locked in the kennels (Figure 2). Serena was one of the few allowed to remain off leash and out of the cage because of her calm temperament. She was elegantly lying in a clover meadow that made both her golden fur and the leishmaniasis lesion on her left forefoot stand out. The ulcer was open and badly swollen, causing her claws to move sideways. Although she must have been in pain, her half‐open eyes seemed to indicate that she was enjoying the morning freshness and the still‐pleasant sunshine (Figure 3).

Serena was about four years old. Her dog handler was Rubén. When both were deemed ready by their trainers, they entered the area of operations and stayed six months there. After a month of rest, as soon as Rubén and Serena met again, she gave him her paw. According to Rubén, she was showing him that something was not quite right. A fungus, he thought. He applied an antifungal cream and Lepecid, a purple antiseptic for cattle and dogs that is also used to kill *nuches* (fly larvae). But none of that worked. On the contrary, it made the lesion even more irritated. Although Rubén asked for permission to stay out of the jungle until Serena's foot had healed, his request was ignored and they had to enter the area of operations again. “Almost three months later, the doggie's lesion got the way it's now, big and raw,” he told me. Still in the jungle, and despite Rubén repeatedly telling his superiors that the sore was likely to be leishmaniasis, he received another antibacterial and antifungal cream. The lesion did not improve, and Serena was suffering from the long walks, branches, puddles, humidity, and accidental stumbling of the soldiers who sometimes inadvertently stepped on her.

A dog like that, with leishmaniasis, in the area, what's the point? It's like having a soldier with appendicitis. In the jungle, he has to walk and suffer. I mean, you suffer for him because you see he's not feeling well. And they [the commanders] start to *mamar gallo* [make up excuses] for not evacuating him. They wait 10, 20 days. They wait until they see it's serious, and then they take him out. This also happens with dogs. A dog doesn't get to be evacuated until they see it *putiado* [broke down] from leishmaniasis. I experienced that with Serena. The doggy had leishmaniasis, they knew it was leishmaniasis, and I was informing the commander it was leishmaniasis, but they didn't take her out of the area. I had to force her to work because, if you don't put the dog to work, they report on you.

Six months passed again until she and Rubén were allowed to leave the jungle. Back in the battalion, Rubén wanted to get an accurate diagnosis for Serena. A bacteriologist at the army dispensary helped him carrying out this procedure and the diagnosis came out positive. What a relief, he thought. However, at that time, Glucantime was no longer authorized for the treatment of army dogs. When I met Serena, she had been treated with other drugs for more than a year and her body had not been able to form a definitive, long‐lasting scar. For Rubén, Serena's tribulations and hardships with leishmaniasis were extremely frustrating and encumbering, as he had not able to work normally since the dog became sick.

“If Serena would get better and the vet decided to take her off duty, would you adopt her?” I asked him, knowing that, in that situation, adoption priority is given to handlers. “Yes! She's so perfectly quiet and obedient, and my son would be so happy with her at home,” he replied. “And if that happens, would you be assigned a different dog?” I asked. “I refuse to accept any more dogs because I've suffered too much with that little dog,” he said. Being in charge of a sick dog with an uncertain prognosis, whose treatment has proven ineffective on several occasions, has been too frustrating for Rubén, so much so that he prefers to stop working as a military dog handler altogether. At the end of that day, Serena was still lying in the clover meadow. From time to time, she would stand up a little, take a couple of limping steps, and settle back into the meadow or a spot out of the sun. If it were up to her, I thought, she would quit her job in the military to start a new life, next to Rubén's son, without leishmaniasis, and far away from the sandflies.

Although the work that dogs like Serena must perform for the military depends on constantly cultivating and nurturing a relationship between them and soldiers, this bond suffers and deteriorates as that same work leads to a breakdown in their health status. When it is no longer clear to what extent a dog can continue to contribute to war, its role and future—as well as that of its companion soldier—are subject to uncertainty and the unlikely effectiveness of some drug designed for a purpose other than leishmaniasis. The treatment of war dogs with leishmaniasis in Colombia shows how their hierarchical status in relation to humans is susceptible to regular change. However, unlike the case analyzed by Hediger (2013), the situation of dogs with leishmaniasis is never fully resolved but, amid an armed conflict that drags on indefinitely, remains suspended in a bureaucratic, medico–scientific, moral, and relational limbo. Also, despite the opinion of soldiers who see themselves and their dogs as indispensable, it is the military and its war efficiency logics that ultimately determine when the time has come to consider the binomial, or one of its halves, disposable.

## A Matter of Susceptibility

Marcela Hoyos was the veterinarian responsible for the medical care of the 500–600 dogs working for the army in the southern *departamentos* ^9^ of Caquetá, Amazonas, and Putumayo. During my visit to the Liborio Mejía Battalion's CERCA, 35 dogs were there: one Golden Retriever, one German Shepherd, and several Labrador Retrievers and Belgian Malinois Shepherds. Despite notorious differences between these breeds, Marcela told me that all dogs were equally affected by leishmaniasis, skin fungi, and tick‐borne (erliquiosis, babesia, and anaplasmosis), respiratory, and digestive diseases. Like soldiers, differences in the susceptibility to leishmaniasis among dogs mostly depended on the area where they were made to work. Where jungles were rare, leishmaniasis cases among dogs (and soldiers) were sporadic. The opposite was true for places where jungles were abundant. Among the 35 dogs I encountered, nine were there for health problems: two for anaplasmosis and seven for leishmaniasis. Marcela told me that times with no dogs with leishmaniasis at the Liborio Mejía battalion were basically nonexistent.

She also explained that, like humans, some dogs were more susceptible to leishmaniasis depending on their immune system and the time they spent in endemic areas:

Dogs’ susceptibility depends on the length of exposure [to infected sandflies] and the response of their immune systems. The literature even reports experiments with dogs that were inoculated with *Leishmania* parasites and didn't develop the disease. That means their immune system was strong enough to defeat the disease. But there are other dogs who aren't like that, who are more susceptible. Some dogs get sick every time they enter the area of operations. So, you see that some dogs end up two or even three times here because of leishmaniasis. Obviously, it's best to avoid sending those dogs back to work. I prefer to put them up for adoption, give them administrative leave so they can leave the army and rest. Why continue sending a dog that is so susceptible to leishmaniasis to an area where it is continuously exposed [to the disease]?

Following this logic, after a maximum of two episodes of leishmaniasis, Marcela removes a dog from service. Her rationale is based on two reasons. First, the dog is seen as a reservoir that potentially represents a risk to public health. Second, it does not make sense for her to keep a dog working in the rainforest if it is going to get sick again. “If you are allergic to dust and have rhinitis, why would I insist that you dust? If I already know that a dog is susceptible to leishmaniasis, why would I put it back in the jungle?” she said.^10^

Once I heard about this policy, I could not help but think of the many soldiers I met who cannot ask for relocation to a nonendemic area after two leishmaniasis episodes. In fact, they have to go through up to five cycles of intoxicating antileishmanial therapy before having the possibility to be reassigned to a different military unit where they are not routinely exposed to sandflies. Soldiers, however, are not automatically relocated after five leishmaniasis episodes. They have to ask for a *junta médica* to take place first—a military occupational medical board that evaluates army members’ acquired disabilities and medically diagnosed conditions. This junta is responsible for quantifying the diminution in the work capacity of an army member and makes decisions on relocation, financial compensation, and permanence in the institution.^11^ In the case of leishmaniasis, it decides the economic compensation soldiers receive for both scar(s)^12^ and conditions accepted as sequelae of treatment (heart, liver, kidney, or infertility problems) only if their symptoms are backed up by medical exams and diagnostic tests.

At the CRL, I was often present at the medical consultations soldiers had to attend before, during, and after their Glucantime treatment. In one of them, a professional soldier asked the doctor about the junta. That was his second leishmaniasis treatment, so he wanted to know if he should ask for his case to be reviewed by such a board. “The army only pays once for any given pathology,” the doctor said. In other words, leishmaniasis scars and treatment sequelae are only compensated once in the military life of a person. If he did ask for a junta to take place at that moment, having passed “only” through two Glucantime treatments, he would have used up his only chance to get any compensation for leishmaniasis. Because he was going to be sent back to the jungle—the doctor continued explaining—it was better for him to wait until he had his fifth antileishmanial treatment. At that moment, not before, he could ask to be relocated to a zone where the probability of getting the disease was very low. So, the doctor recommended him to wait until the fifth treatment to request his case to be evaluated by the junta. At that point, the medical board would probably decide to compensate him for *all* the accumulated scars and treatment sequelae from *all five* Glucantime treatments. “*Véalo como un ahorro* [look at it as savings],” the doctor brutally concluded. As these cruel words were spoken, I thought of the contrasting experience of military dogs with leishmanaisis who were spared three of these five cycles of Glucantime poisoning on a regular basis.

Although dogs and soldiers are similarly susceptible to the disease, the chances of avoiding new episodes of leishmaniasis and new cycles of antileishmanial therapy are very different for human and nonhuman members of the army. While dogs are withdrawn from service after two episodes of leishmaniasis, soldiers can only request relocation to a nonendemic area after five leishmaniasis treatments. In addition, the army compensates soldiers who have gone through the hardships of leishmaniasis only once. This system is perverse. It encourages the few soldiers who are aware of the leishmaniasis‐related financial compensations, to which they are entitled but often uninformed on, to stay in the military, putting their health and youth at the service of war despite the disease and the harms that each course of treatment entails. Army dogs also suffer from the instrumentalization of war, disposed of after they are no longer usable on the battlefield. Yet, they are better off than soldiers when they are spared repeated suffering due to leishmaniasis. As such, the typical hierarchies between humans and animals are once again reversed.

## Conclusion

Reflecting on recent humanitarian and health frameworks that do not focus primarily on the well‐being of humans, but on that of nonhuman animals, the environment, and the planet more generally, Ticktin asks: “Whose health matters, how do we conceive of its boundaries—by way of affective ties, political connections, or biological measurements—and how do these criteria get combined?” (2019: 135). Drawing inspiration from these words, in this article I have shown that war, its violence, the affects it exploits, and the biopolitical logics it employs play a crucial role in how canine binomials of the Colombian army are crafted, how intra‐ and interspecies hierarchies emerge and shift, and how the health of military humans and dogs—as well as that of civilian humans and dogs—is or is not cared for. Both health and war have a more‐than‐human nature, which is reflected in the biopolitical management of humans and dogs with leishmanaisisis inside and outside the military. Although the distinctions and resulting hierarchies are unstable and not always anthropocentric (humans are not always above military dogs), various forms of suffering for humans, dogs, and the interspecies bond that intensifies with each passing day in the war, are constantly produced.

The experience of leishmaniasis, shared by dogs and humans, points to the need for public health—and peace‐building efforts—to articulate and reconsider suffering as a more‐than‐human capacity in terms of both disease and war. As Nading discusses, “a shared capacity to suffer—to feel pain and discomfort bodily, rather than to express it linguistically—forges a moral connection between humans and other animals” (2013: 71). In that sense, documenting, recognizing, and repairing the consequences of war also means understanding health afflictions as inherent to the armed conflict, and the human as part of a heterogeneous group of beings who have suffered it. In other words, the experiences of dogs and humans with leishmaniasis in (post‐)conflict Colombia invite us to conceive and implement new and nonviolent practices of care that, drawing on feminist thinking, “can be retooled to address persistent forms of exclusion and domination” that arise from war and have continued despite the signing of a peace agreement (Ticktin 2019: 136; see also Pinto‐García 2019).

Although the health problem posed by military dogs with leishmaniasis is not usually solved by euthanizing them, MinSalud does consider that killing them is the way to address the issue in the civilian and military spheres. However, this article makes the case that we need to start moving toward health policymaking that involves a contextual discussion about dependency relationships, affective ties, and the distribution of damages and benefits between human and canine populations (Rock et al. 2017). Decisions about how to address leishmaniasis among military dogs cannot ignore the life‐saving role these animals have played in Colombia's armed conflict, the affects and emotions that constitute the canine binomial, and the vulnerabilities to which the state continues to expose them to. Likewise, the necessary changes in the way leishmaniasis is addressed for other populations, both human and canine, also require a political reimaginaing of health care that very consciously moves away from the war‐imposed aims and valuations of human and nonhuman life.

In the challenging moment Colombia is experiencing, where the achievement of a peace agreement is under daily jeopardy, situations such as that of military dogs with leishmaniasis highlight how necessary a more‐than‐human perspective may be to turn health care into a peace intervention. It involves repairing, through a set of historically and politically situated practices and affects, all the relationships that war and violence have sickened, perverted, or destroyed, including those between humans and nonhumans. It requires us to identify, intervene, and address the violence that persists in the health provisions despite the peace deal, which are problematic not necessarily because of their anthropocentric nature, but because of the harm they cause to humans, nonhumans, and the bonds between them.

## Notes

1 All the names in this article, whether human or canine, are pseudonyms.

2 In this article, the word “army” is restricted to the Colombian state military forces and the word “soldier” to first‐line combat members of this institution.

3 I use the word “leishmaniasis” to refer exclusively to cutaneous leishmaniasis.

4 In conversations with former guerrilla members, I learned that opossums and rats that inhabit the jungle can also carry leishmaniasis ulcers.

5 Definition from the Oxford's Spanish dictionary.

6 For dogs, a dose of 75–100 mg of meglumine antimoniate (Glucantime's active ingredient) per kilogram of body weight for 30 days used to be employed.

7 The same is true for any other antileishmanial drugs because all of them exclusively circulate through paths established by MinSalud for the health care of humans, not animals. Glucantime is the first‐line antileishmanial drug in Colombia. If this drug fails, the state recommends the use of other pharmaceuticals (miltefosine, pentamidine, or amphotericin B) which are also highly toxic. If it is already difficult to access Glucantime, it is even more challenging to access any of these second‐line drugs.

8 There are asymptomatic patients, that is, dogs who do not develop ulcers despite having parasites circulating in their bodies. The same goes for humans.

9 Colombia is administratively divided into 32 departamentos.

10 Jaime Rivera, at CENAM, confirmed that military dogs are usually removed from service after two leishmaniasis treatments.

11 See Decree 1796 of 2000.

12 The economic compensation is calculated based on a classification and score system established by the Decree 0094 of 1989. Leishmaniasis scars on the face, for example, have a higher score—involve a higher compensation—than leishmaniasis scars located on other parts of the body.
